# T Cell Specific Adapter Protein (TSAd) Interacts with Tec Kinase ITK to Promote CXCL12 Induced Migration of Human and Murine T Cells

**DOI:** 10.1371/journal.pone.0009761

**Published:** 2010-03-18

**Authors:** Tone Berge, Vibeke Sundvold-Gjerstad, Stine Granum, Thorny C. B. Andersen, Gunn B. Holthe, Lena Claesson-Welsh, Amy H. Andreotti, Marit Inngjerdingen, Anne Spurkland

**Affiliations:** 1 Department of Anatomy, Institute of Basic Medical Sciences, University of Oslo, Oslo, Norway; 2 Department of Genetics and Pathology, Rudbeck Laboratory, Uppsala University, Uppsala, Sweden; 3 Department of Biochemistry, Biophysics and Molecular Biology, Iowa State University, Ames, Iowa, United States of America; University Paris Sud, France

## Abstract

**Background:**

The chemokine CXCL12/SDF-1α interacts with its G-protein coupled receptor CXCR4 to induce migration of lymphoid and endothelial cells. T cell specific adapter protein (TSAd) has been found to promote migration of Jurkat T cells through interaction with the G protein β subunit. However, the molecular mechanisms for how TSAd influences cellular migration have not been characterized in detail.

**Principal Findings:**

We show that TSAd is required for tyrosine phosphorylation of the Lck substrate IL2-inducible T cell kinase (Itk). Presence of Itk Y511 was necessary to boost TSAd's effect on CXCL12 induced migration of Jurkat T cells. In addition, TSAd's ability to promote CXCL12-induced actin polymerization and migration of Jurkat T lymphocytes was dependent on the Itk-interaction site in the proline-rich region of TSAd. Furthermore, TSAd-deficient murine thymocytes failed to respond to CXCL12 with increased Itk phosphorylation, and displayed reduced actin polymerization and cell migration responses.

**Conclusion:**

We propose that TSAd, through its interaction with both Itk and Lck, primes Itk for Lck mediated phosphorylation and thereby regulates CXCL12 induced T cell migration and actin cytoskeleton rearrangements.

## Introduction

The CXC chemokine subfamily member, CXCL12/stromal cell-derived factor (SDF)-1α, is expressed in a broad range of tissues and has multiple effects on lymphoid and endothelial cells (reviewed in [1]). Mice deficient in either CXCL12 or its seven-transmembrane G protein coupled receptor (GPCR) CXCR4 die perinatally and display profound defects in the hematopoietic system [2], [3]. CXCL12 modulates T cell development in the thymus [4], [5], T cell adhesion and migration [6], as well as expression of genes controlling T cell signaling, migration and survival [7]. These effects are mediated through multiple signaling pathways, including the Ras, ERK [8], the JAK/STAT [9] and the PI3K-1A and -1B pathways [10], [11]. In addition, CXCL12 has been reported to co-stimulate activation of T cells [12] by promoting a physical association between CXCR4 and the T cell receptor (TCR) [13].

T cell specific adapter protein (TSAd) [14] (also known as Lck-associated adapter protein (LAD) [15], Rlk/Itk binding protein (RIBP) [16] and vascular endothelial growth factor receptor adapter protein (VRAP) [17]), is encoded by the *SH2D2A* gene, and its expression is rapidly induced in human T cells upon TCR triggering [14], [16], [18], [19]. TSAd contains a Src homology (SH) 2 domain, a proline-rich region with potential SH3 interaction sites, as well as several tyrosine phosphorylation sites [14], [20]. In T cells, TSAd interacts with and becomes tyrosine phosphorylated by the Src kinase Lck [15], [20], enabling TSAd to modulate Lck function and thereby influence downstream TCR signaling events [18], [20]–[23]. TSAd is also expressed in endothelial cells, where it docks onto activated vascular endothelial growth factor receptor 2 (VEGFR-2) and promotes actin stress fiber formation and migration of endothelial cells [24]. Recently, Park and colleagues showed that TSAd, through association with the G-protein β subunit, regulates chemokine-dependent migration of Jurkat T cells [25].

The Tec family kinase IL-2 inducible T-cell tyrosine kinase (Itk) has been identified as a TSAd interaction partner in a yeast two-hybrid screen [16]. Itk regulates CXCL12 induced activation of Rho GTPases, cell polarization, adhesion and migration of T cells [26]–[28]. Furthermore, Itk is tyrosine phosphorylated in response to CXCL12 [26], [27] in a Src-kinase-dependent manner [26]. Lck-mediated tyrosine phosphorylation of Itk is necessary for activation of its kinase activity [29]. In agreement with this, Lck is activated in response to CXCL12 [30], [31] and kinase activities of both Itk [27] and Lck [30] are important for CXCL12 mediated T-cell chemotaxis. Moreover, Lck has recently been shown to be recruited to TSAd upon CXCL12 stimulation of Jurkat T cells [25].

Although both Itk and TSAd have been implied in chemokine induced cellular migration, the role of the TSAd-Itk interaction in cellular migration has not previously been addressed. Here we show that the interaction between TSAd and Itk depends on the C-terminal proline-rich region of TSAd (aa239-274) and the SH3 domain of Itk. Using Jurkat T cells as a model system, we show that the positive effect of TSAd on CXCL12 induced chemotaxis is dependent on its Itk interaction site and on an intact Itk Y511 phosphorylation site. In accordance with this, CXCL12 stimulation of TSAd deficient murine thymocytes failed to induce tyrosine phosphorylation of Itk, and these cells also displayed reduced CXCL12-induced actin polymerization and chemotaxis. Together, our data indicate that TSAd regulates CXCL12 induced T-cell migration and actin cytoskeleton rearrangements by promoting Lck dependent phosphorylation of Itk.

## Results

### TSAd interacts through its proline rich region with the Itk SH3 domain

We previously found that TSAd is important for VEGF-induced actin stress-fiber formation and migration of endothelial cells [24]. Furthermore, TSAd was recently found to promote chemokine induced migration of Jurkat T cells [25]. Similarly, the TSAd interaction partner Itk [16] has been found to be involved in CXCL12 induced actin polymerization and T cell migration [26], [27]. However, it is unknown whether the interaction of TSAd with Itk is required for TSAd's effect on chemokine induced cellular migration. To analyze this, we first set out to map the interaction site of TSAd with Itk.

**Figure 1:**
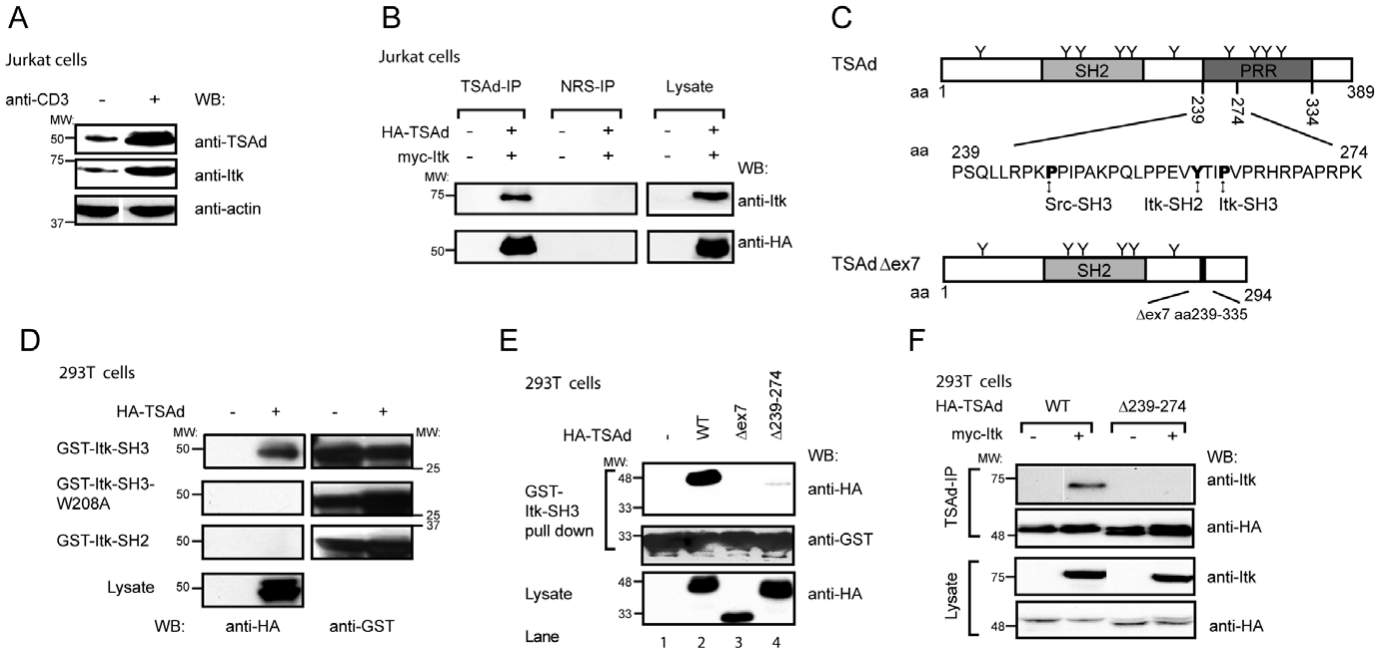
The C-terminal proline-rich region of TSAd interacts with the Itk-SH3 domain. (A) Jurkat cells were kept unstimulated or stimulated with anti-CD3 for 22 hours. Lysates were immunoblotted with the indicated antibodies. (B) Lysates from Jurkat T cells expressing myc-tagged Itk together with HA-tagged TSAd were subjected to immunoprecipitation with anti-TSAd antibody or normal rabbit serum (NRS) as negative control. The precipitates were immunoblotted with anti-HA and anti-Itk antibodies. Cell lysates were immunoblotted with the same antibodies to verify expression from transfected plasmids. (C) A schematic drawing of TSAd and TSAd Δex7 with their protein domains and potential tyrosine phosphorylation sites (Y) included. The region removed in the TSAd Δ239–274 deletion mutant is depicted by amino acid positions in the upper part, and its sequence is shown in the lower part. The P ( =  proline) and Y ( =  tyrosine) in bold mark the proposed interaction sites for the Src-SH3, Itk-SH3 and Itk-SH2 domains. SH2  =  Src homology 2 domain, SH3  =  Src homology 3 domain, PRR  =  proline-rich region. (D) Lysates from 293T cells with or without HA-TSAd expression were subjected to pull down experiments using the indicated GST-fusion proteins. Presence of pulled down HA-tagged TSAd was demonstrated by immunoblotting with anti-HA-antibody. The amount of GST-fusion proteins used was verified by immunoblotting with anti-GST antibody. Cell lysates were immunoblotted with anti-HA antibody to show expression of HA-tagged TSAd. (E) Lysates of 293T cells transfected with empty vector or plasmids encoding HA-tagged wild type TSAd (WT) or TSAd mutant proteins, i.e. TSAd Δex7 or TSAd Δ239–274 were incubated with GST-Itk-SH3. Pulled down proteins and cell lysates were analyzed as in D. (F) Lysates from 293T cells expressing wild type TSAd or TSAd Δ239–274 alone or together with myc-Itk were subjected to immunoprecipitation with anti-TSAd antibody. Precipitates and corresponding lysates were analyzed as in B.

Murine TSAd has previously been shown to interact with Itk in yeast and in transiently transfected HEK293 cells [16]. We therefore first aimed to extend this initial observation of the TSAd-Itk interaction to also include T cells. Itk and TSAd are expressed at low levels in resting primary T cells, but are induced upon activation of T cells through the TCR [14], [32], [33]. The same is true for Jurkat T cells (Fig. 1A). In contrast, CXCL12 does not induce expression of either TSAd or Itk [7], [34]. Since we here focused on the possible functional link between TSAd and Itk in CXCL12/CXCR4 signaling, and since TCR stimulation may affect subsequent CXCR4 signaling [35], we chose to use over expression of TSAd and Itk in Jurkat T cells for most of our studies. TSAd was immunoprecipitated from lysates of unstimulated Jurkat T cells transiently expressing human HA-tagged TSAd and human myc-tagged Itk. As shown in Fig. 1B, Itk could be co-immunoprecipitated with anti-TSAd antibody, but not with normal rabbit serum (NRS), indicating that human Itk and TSAd are able to interact also in T cells.

*In silico* analysis revealed that the C terminus of TSAd contains possible interaction sites for both the SH2 and the SH3 domains of Itk (http://scansite.mit.edu/) (Fig. 1C). To further dissect the Itk-TSAd interaction, we thus performed glutathione-S-transferase (GST) pull-down experiments using GST-Itk-SH2 and GST-Itk-SH3 fusion proteins in cell lysates from 293T cells transiently expressing TSAd. GST-Itk-SH3 mutated for tryptophan 208 (GST-Itk-SH3-W208A), which is crucial for binding of polyproline motives, was included as a negative control. TSAd was pulled down with GST-Itk-SH3, but not with GST-Itk-SH3-W208A, GST-Itk-SH2 (Fig. 1D) or GST (data not shown and [21]). SH2 domain interactions are dependent on tyrosine phosphorylation of the ligands, however GST-Itk-SH2 also failed to interact with tyrosine phosphorylated TSAd expressed in the presence of Lck (data not shown and [21]). SH3 pull-down experiments in lysates from 293T cells transiently expressing TSAd with a deletion of the C-terminal exon 7 (TSAd Δex7, Fig. 1C), revealed that the removal of the C-teminal part of TSAd totally abolished its interaction with Itk-SH3 (Fig. 1E, lane 3). Only a very faint band of TSAd deleted for the putative Itk SH3 interaction site aa 239–274 (TSAd Δ239–274) was detected on longer exposures after Itk-SH3 pull down of (Fig. 1E, lane 4 and data not shown). These results were supported by experiments in transiently transfected 293T cells, where myc-tagged Itk was found to co-immunoprecipitate with TSAd but not with TSAd Δ239–274 (Fig. 1F). Taken together, these data show that Itk interacts with TSAd via its SH3 domain, and that TSAd amino acids 239–274 are crucial for the interaction with Itk.

### The proline-rich region of TSAd influences CXCL12 induced actin polymerization and migration

**Figure 2:**
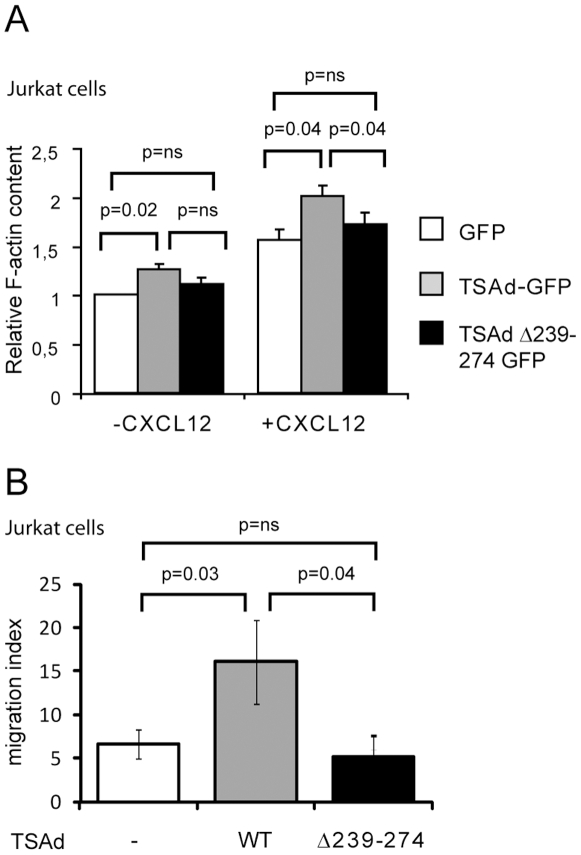
The proline-rich region of TSAd is important for CXCL12 induced actin polymerization and migration. (A) Jurkat T cells transiently transfected with plasmids encoding GFP (−), or GFP fused to wild type TSAd or TSAd Δ239–274 were left unstimulated (-CXCL12) or stimulated with 100 ng/ml CXCL12 for 15 seconds prior to staining of F actin with Alexa 546-phalloidin followed by flow cytometry analysis. The F-actin content was assigned as mean fluorescence intensity value of FITC-phalloidin stained cells. Data shown are F-actin content relative to F actin in unstimulated cells expressing GFP. Mean values +/− standard error of the mean (SEM) from four independent experiments are shown. (B) Jurkat T cells were transfected as in A with plasmids encoding GFP and TSAd, either in fusion or as separate proteins, and assayed for migration towards 100 ng/ml CXCL12. Migration index was calculated as the number of GFP-positive cells migrating in response to CXCL12 divided by GFP-positive cells migrating towards medium only. The mean migration indices of four independent experiments with SEM are shown.

We next examined whether interaction of TSAd with Itk influences actin polymerization in response to CXCL12 stimulation of Jurkat T cells transiently expressing GFP alone, or GFP fused to TSAd or to TSAd Δ239–274. Flow cytometry analysis revealed that both resting and CXCL12 stimulated cells expressing intact TSAd displayed significantly higher levels of F-actin than cells expressing GFP alone (Fig. 2A). By contrast, resting and CXCL12 stimulated cells expressing TSAd Δ239–274 displayed non significant increases in F-actin level compared with cells expressing GFP alone (Fig. 2A). Moreover, in accordance with Park's previous report, we found that Jurkat T cells expressing TSAd displayed significantly higher migratory response upon CXCL12 stimulation compared to Jurkat cells expressing GFP alone, whereas cells expressing TSAd Δ239–274 showed a chemotactic response comparable to that of GFP-expressing cells (−) (Fig. 2B). Taken together, these data suggest that TSAd promotes CXCL12 induced actin polymerization, as well as migration of Jurkat T cells. Moreover, our results indicate that the proline rich region (aa 239–274) of TSAd and thus potentially TSAd's interaction with Itk, is crucial for this effect.

### TSAd promotes tyrosine phosphorylation of Itk on Y511

In Jurkat T cells, CXCL12 induces Src kinase dependent tyrosine phosphorylation of Itk [26], [27] and Lck (or Src)-mediated phosphorylation of Y511 in the kinase domain of Itk is essential for its activation in primary T cells upon TCR stimulation [29], [36]. TSAd is known to interact with the Src kinase Lck [15] through the Lck SH2- and SH3-domains [22] via multiple interaction sites [20]. Furthermore, Park and colleagues recently reported that TSAd associates with Lck upon CXCL12 stimulation of Jurkat T cells [25]. However, whether the TSAd-Lck interaction was required for TSAd's effect on chemokine stimulation was not addressed.

**Figure 3:**
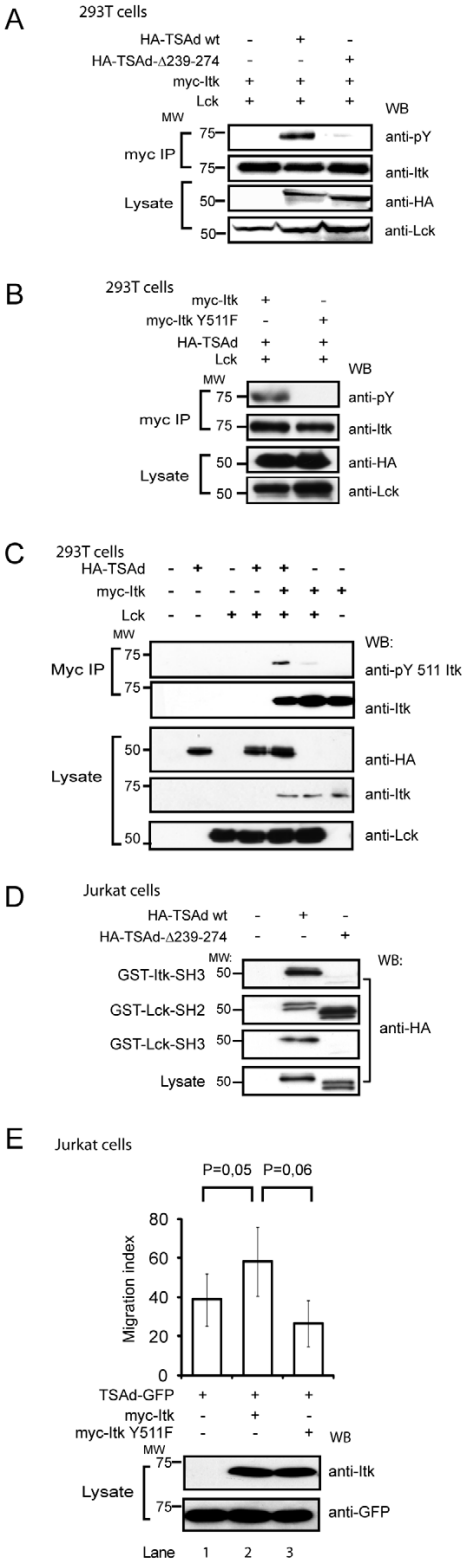
TSAd promotes Lck mediated tyrosine phosphorylation of Itk. (A) Itk was immunoprecipitated with an anti-myc antibody from 293T lysates expressing the indicated proteins and immunoblotted with anti-phosphotyrosine antibody. The blot was stripped and reprobed with anti-Itk antibody to ensure equal immunoprecipitation. The corresponding lysates were probed with the indicated antibodies to verify expression from the transfected plasmids. (B) As in A. (C) Myc-Itk was immunoprecipitated from lysates of 293T cells transfected with plasmids encoding the indicated proteins and immunoprecipitates were immunoblotted with an antibody specific for tyrosine-phosphorylated Itk Y511. Immunprecipitates and lysates were also immunoblotted with the indicated antibodies to verify equal immunoprecipitation efficiency and expression from transfected plasmids. (D) Lysates from Jurkat T cells expressing wild type TSAd or TSAd Δ239–274 were incubated with the indicated GST-fusion proteins. Presence of pulled down HA-tagged TSAd as well as HA-tagged TSAd in cell lysates were demonstrated by immunoblotting with anti-HA-antibody. (E) Jurkat T cells transiently transfected with plasmids encoding the indicated proteins were assayed for migration towards 100 ng/ml CXCL12. Migration index was calculated as the number of GFP-positive cells migrating in response to CXCL12 divided by GFP-positive cells migrating towards medium only. Mean values +/− SEM from three independent experiments are shown. Western blots verify expression from transfected plasmids in one of the experiments.

Since Lck may phosphorylate Itk [29], and since TSAd interacts both with Itk and Lck, we first examined whether TSAd influences Lck mediated tyrosine phosphorylation of Itk. In 293T cells, Itk was tyrosine-phosphorylated when co expressed with Lck and TSAd, however no Itk phosphorylation was observed when Itk was co-expressed with Lck and TSAd Δ239–274 (Fig. 3A). Furthermore Lck was unable to phosphorylate the Itk Y511F mutant in the presence of TSAd (Fig. 3B). When Myc-tagged Itk was immunoprecipitated from 293T cells using a Myc antibody, a band corresponding to Myc-Itk was detected by anti-pY551 Btk antibody (which also recognize phosphorylated Y511 Itk) only in cells co-expressing TSAd, Lck and Itk (Fig. 3C). Taken together these results show that phosphorylation of Y511 Itk in 293T cells requires the presence of both TSAd and Lck.

The amino acids 239–274 of TSAd affects interaction not only with the Itk-SH3 domain (Fig. 1E and F) but also with the SH3 domain of Lck [22], as Lck-SH3 binds to the Scansite-predicted Src-SH3 site, see Fig. 1C. However, in contrast to Itk, Lck can also interact with several other sites within TSAd [22], suggesting that Lck should still be able to bind to the truncated TSAd Δ239-274 protein. This notion was directly tested by additional pull-down experiments in Jurkat T cell lysates. Neither Itk-SH3 nor Lck-SH3 interacted with TSAd Δ239–274, whereas the Lck-SH2 domain could pull down this mutant (Fig. 3D). Note that Lck-SH3 and Itk-SH3 do not discriminate between different phosphorylated forms of TSAd. Thus, the two SH3 pull down experiments display the same ratio of the two migrating bands as seen in the lysates, whereas Lck-SH2 preferentially binds to the slowest migrating TSAd band, probably representing a phosphorylated form of TSAd. Taken together, these results suggest that the lack of Itk tyrosine phosphorylation in the presence of Lck and TSAd Δ239–274 (Fig 3A) is likely due to the loss of the interaction between Itk and the deleted TSAd mutant protein.

It has previously been shown that transient over-expression of Itk in Jurkat cells promotes chemokine induced migration [27]. The scarce expression of endogenous Itk in resting Jurkat T cells (Fig. 1A), suggests that endogenous Itk levels may be a limiting factor when transiently expressing TSAd alone. We therefore assayed migration of Jurkat T cells that were transfected with plasmids encoding Itk and GFP-TSAd. The migratory response of Jurkat cells co-expressing Itk and GFP-TSAd upon CXCL12 stimulation was approximately 50% higher than that observed for cells expressing GFP-TSAd alone (Fig. 3E). The positive effect on CXCL12 induced chemotaxis was dependent upon Itk Y511, since cells co-expressing GFP-TSAd with Itk Y511F displayed similar or even lower migratory response than cells expressing GFP-TSAd alone (Fig. 3E). Taken together, these observations suggest that TSAd interacts with Itk to enhance Lck mediated phosphorylation of Itk Y511 and that phosphorylation of this residue is important for TSAd's positive effect on chemokine induced migration.

### CXCL12 induced Itk tyrosine phosphorylation is dependent on TSAd in T cells

**Figure 4:**
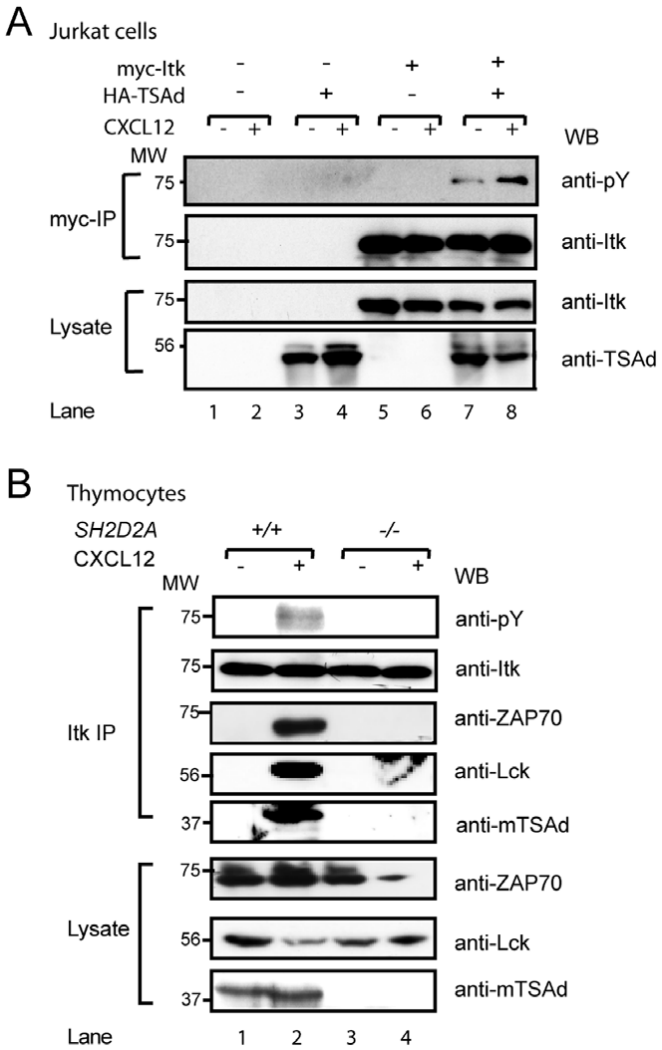
CXCL12 mediated Itk tyrosine phosphorylation is dependent on TSAd expression in T cells. (A) Jurkat T cells transiently transfected with plasmids encoding the indicated proteins were kept unstimulated (−) or stimulated (+) with 500 ng/ml CXCL12 for 2 min and then lysed. Itk was immunoprecipitated with anti-myc antibody and immunoblotted with anti-phosphotyrosine antibody. The blot was stripped and reprobed with anti-Itk antibody to ensure equal immunoprecipitation. The corresponding lysates were probed with the indicated antibodies to verify expression from the transfected plasmids. (B) Thymocytes from *SH2D2A* ^+/+^ or *SH2D2A* ^−/−^ mice were stimulated with 100 ng/ml CXCL12 for 2 minutes (+), lysed and subjected to immunoprecipitation with anti-Itk antibody. Immunoprecipitates were subjected to immunoblotting with anti-phosphotyrosine antibody to detect tyrosine phosphorylated Itk. The blot was stripped and reprobed with anti-Itk antibody to ensure equal immunoprecipitation efficiency, and with anti-Lck, anti-Zap70 and anti-mTSAd (murine TSAd) antibodies to detect co-immunoprecipitation of these proteins with Itk. Lysates were probed with anti-mTSAd, anti-Lck and anti-Zap70 antibodies to verify their expression.

To examine whether CXCL12 induced tyrosine phosphorylation of Itk is influenced by TSAd expression in T cells, Jurkat T cells transiently transfected with plasmids encoding HA-TSAd and/or myc-Itk were stimulated with CXCL12, and tyrosine phosphorylation of immunoprecipitated myc-Itk was assessed (Fig. 4A). Exogenously expressed Itk was weakly tyrosine phosphorylated in Jurkat cells when expressed alone (seen in longer exposures of the anti-pY blot in Fig. 4A, data not shown). Co-expression of TSAd increased the phosphorylation level of Itk substantially, particularly in CXCL12 stimulated cells (Fig 4A, compare lanes 7 and 8 to lanes 5 and 6), supporting the notion that TSAd promotes phosphorylation of Itk.

To examine whether TSAd influences phosphorylation of Itk also in primary cells we performed experiments in thymocytes from mice lacking TSAd. To the best of our knowledge, these cells are the only primary cells that express both TSAd [14] and Itk [32] at relatively high levels without prior stimulation. Moreover, thymocytes express CXCR4 and are responsive to CXCL12 stimulation [37]. In accordance with our findings in Jurkat cells transiently expressing TSAd and Itk, CXCL12 stimulation induced Itk tyrosine phosphorylation in thymocytes from wild type *SH2D2A* ^+/+^ mice, but not in thymocytes from *SH2D2A* ^−/−^ mice (Fig. 4B, upper blot).

Park *et al* [25] have previously shown that upon CXCL12 stimulation, TSAd associates with Lck and Zap-70 in Jurkat T cells expressing TSAd. In line with these findings, we found that Lck and Zap-70 could also be co-immunoprecipitated with Itk and TSAd from normal CXCL12 stimulated thymocytes but not from *SH2D2A* ^−/−^ thymocytes (Fig. 4B, middle blots). This indicates that Lck and Zap-70 are recruited to Itk in a TSAd-dependent manner upon CXCL12 stimulation also in primary cells.

### Actin polymerization and cellular chemotaxis are impaired in SH2D2A^−/−^ thymocytes

**Figure 5:**
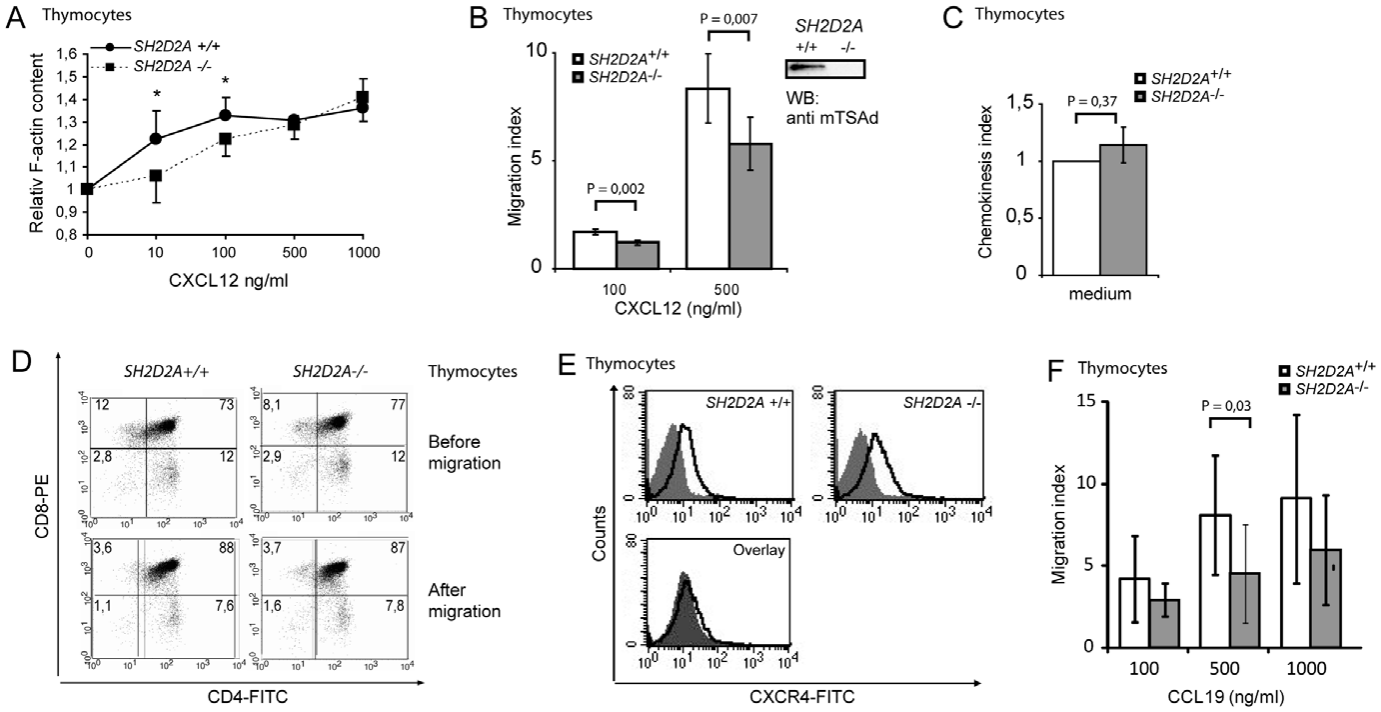
CXCL12 mediated actin polymerization and cellular migration is impaired in murine TSAd-deficient thymocytes. (A) Thymocytes from *SH2D2A* ^+/+^ or *SH2D2A* ^−/−^ mice were left unstimulated (0) or stimulated with 10, 100, 500 or 1000 ng/ml CXCL12 for 15 seconds and stained for F actin. The F-actin content was assigned as mean fluorescence intensity value of FITC-phalloidin stained cells. Data shown are F-actin content after 15 seconds of stimulation with the indicated CXCL12 concentrations relative to F-actin content in unstimulated cells. The graph represents the mean value from at least four independent experiments, +- SEM, *  =  P<0,05. (B) Migration of thymocytes from *SH2D2A* ^+/+^ or *SH2D2A* ^−/−^ mice in response to medium alone or indicated amounts of CXCL12 was assessed using a transwell migration assay. Migration index (MI) was calculated as the number of cells migrating towards CXCL12 relative to the number of cells migrating towards medium only. The graph shows mean MI values +/− SEM of at least four independent assays. The Western blot verifies expression of murine TSAd (mTSAd). (C) Chemokinesis index is calculated as spontaneous migration of either *SH2D2A^+/+^* or *SH2D2A^−/−^* thymocytes relative to that observed for *SH2D2A* ^+/+^ thymocytes in each independent experiment presented in B. The graph shows mean chemokinesis index values +/− SEM of eight independent assays. (D) *SH2D2A* ^+/+^and *SH2D2A* ^−/−^ thymocytes prior to (top panels) or after (lower panels) CXCL12 induced migration were stained with fluorescently labeled anti-CD4 and CD8 antibodies and analyzed by flow cytometry. To achieve sufficient cells for fluorescently labeling after migration, 4×10^6^ thymocytes were allowed to migrate for 4 hours prior to staining. (E) Thymocytes from *SH2D2A* ^+/+^ and *SH2D2A* ^−/−^ mice were analyzed for CXCR4 expression by flow cytometry. Left and middle panel: grey areas - control antibody staining; open areas - CXCR4 staining, right panel: grey areas - CXCR4 staining of *SH2D2A* ^+/+^ cells, open areas – CXCR4 staining of *SH2D2A* ^−/−^ cells. (F) Migration of thymocytes from *SH2D2A* ^+/+^ or *SH2D2A* ^−/−^ mice in response to medium alone or indicated amounts of CCL19 was assessed using a transwell migration assay. MI was calculated as in panel 5B. The graph shows mean MI values +/− SEM of at least three independent assays.

To assess whether TSAd also affects CXCL12 induced actin polymerization in primary cells, thymocytes from normal and TSAd deficient (*SH2D2A^−/−^*) mice were stimulated with various concentrations of CXCL12 for different time periods (15 sec to 1 hr), and FITC-phalloidin stained F-actin was measured by flow cytometry. The basal F-actin level was lower in *SH2D2A* ^−/−^ thymocytes relative to *SH2D2A* ^+/+^ thymocytes, and in both cell types F-actin values peaked after 15 seconds of stimulation for all CXCL12 concentrations tested (data not shown). Upon stimulation with low to moderate concentrations of CXCL12 (10 and 100 ng/ml), the relative increase in F-actin content after 15 seconds of stimulation was significantly higher in *SH2D2A^+/+^* compared to *SH2D2A* ^−/−^ thymocytes (Fig. 5A). However, upon addition of higher CXCL12 doses, the requirement for TSAd seems to be overcome leading to F-actin levels in *SH2D2A* ^−/−^ thymocytes that are similar to that of *SH2D2A^+/+^* cells (Fig. 5A).

We then examined whether CXCL12 induced migration of primary cells was affected by TSAd expression using a transwell chemotaxis assay. Thymocytes from *SH2D2A* ^+/+^ and *SH2D2A* ^−/−^ mice were unresponsive to 10 ng/ml CXCL12 (data not shown), whereas 100 and 500 ng/ml of CXCL12 induced significantly greater migration of *SH2D2A^+/+^* than *SH2D2A^−/−^* thymocytes (Fig. 5B). This was not due to difference in the spontaneous migration of the thymocytes (i.e. chemokinesis) (Fig. 5C). At even higher concentrations of CXCL12 (1000 ng/ml) no difference in migration between *SH2D2A^+/+^* and *SH2D2A^−/−^* thymocytes was observed (data not shown). Analysis of CD4 and CD8 expression of migrating versus non-migrating thymocytes revealed that both for the *SH2D2A* ^+/+^ and the *SH2D2A* ^−/−^ thymocytes, double positive cells were the predominant CXCL12 responders (Fig. 5D). The reduced responsiveness of the *SH2D2A* ^−/−^ cells could not be explained by reduced expression of the CXCL12 receptor CXCR4, as *SH2D2A* ^−/−^cells expressed equivalent levels of CXCR4 compared to *SH2D2A* ^+/+^ cells (Fig. 5E).

Since TSAd has been shown to interact with the Gβ protein [25], it probably affects signalling downstream of several GPCRs in addition to CXCR4. We therefore tested whether TSAd also affected migration towards CCL19, as the CCL19 receptor, CCR7, is expressed on all naïve T cells [38] as well as on thymocytes undergoing positive selection [39]. Addition of 500 ng/ml CCL19 induced significantly greater migration of *SH2D2A^+/+^* compared to *SH2D2A^−/−^* thymocytes (Fig. 5F), while expression of CCR7 was comparable in *SH2D2A^+/+^* and *SH2D2A^−/−^* thymocytes (data not shown).

Taken together, our data show that lack of TSAd in primary cells attenuates actin polymerization and cellular migration upon CXCL12 stimulation, and that TSAd promotes CXCL12 signalling through its interaction with Itk, possibly by priming Itk for tyrosine phosphorylation by Lck. This positive effect of TSAd on chemokine induced migration extends also to CCL19, suggesting that TSAd may play a more general role in GPCR signalling.

## Discussion

In this study we provide evidence that CXCL12 signaling is regulated by a novel molecular mechanism, involving the interaction between TSAd and Itk. We propose that TSAd primes Itk for Lck mediated phosphorylation, and thereby modulates CXCL12 mediated T cell migration and actin polymerization.

The interaction between TSAd, Itk and Lck was demonstrated both in over expression systems in cell lines and in primary cells. In transfected 293T and Jurkat T cells the interaction between TSAd and Itk, and phosphorylation of Itk was observed in the absence of exogenous stimuli, whereas in primary cells the TSAd-Itk-Lck complex formation and Itk phosphorylation was only observed upon CXCL12 stimulation. This apparent discrepancy could be due to high expression levels of the interacting proteins in transfected cell lines, which may overcome the requirement for exogenous stimuli to induce the formation of protein complexes. Moreover, in primary cells, Itk is recruited to the cell membrane via its PH domain after activation of the PI3 kinase [40], whereas in Jurkat T cells a large proportion of Itk is constitutively associated with the cell membrane due to lack of the phosphatase PTEN [41]. This difference between primary cells and Jurkat T cells could explain why we observe some tyrosine phosphorylated Itk also in resting Jurkat cells expressing TSAd.

TSAd was previously identified as an Itk interaction partner in a yeast two hybrid screen [16]. Here we show for the first time that this interaction occurs between the Itk-SH3 domain and the proline-rich C terminus of TSAd (aa239–274) and that this interaction is crucial both for Lck mediated phosphorylation of Itk, as well as for TSAd's effect on T cell migration and actin polymerization. This proline-rich sequence of TSAd also contains an Lck-SH3 interaction site [22], but since Lck can interact with TSAd also outside this proline-rich region ([20], [22], [23] and Fig. 3B), we consider it most likely that the reduced T cell migration and actin polymerization responsiveness upon CXCL12 stimulation in cells expressing TSAd Δ239–274 can be explained by the loss of the interaction with Itk rather than Lck. In support of this notion is the observation that thymocytes from TSAd-deficient mice and Itk-deficient mice display similar relative reductions in migration and actin polymerization [26]. However, we cannot presently exclude that other proteins interact with TSAd through its proline-rich sequence, thereby contributing to TSAd's effect on actin polymerization and cell migration.

Chemokine induced migration and actin polymerization in T cells requires Itk kinase activity [27]. How CXCL12 mediates activation of Itk is still only partially known. To become active, Itk is recruited to the membrane upon cell surface receptor stimulation (reviewed in [28]) followed by tyrosine phosphorylation of Y511 in its kinase domain. In Jurkat T cells, Itk is tyrosine phosphorylated in a Src-kinase-dependent fashion upon CXCL12 treatment [26]. We and others have previously shown that TSAd modulates TCR mediated signaling through interaction with Lck [15], [18], [20], [21], [23]. Moreover, Lck is recruited to TSAd upon CXCL12 stimulation in Jurkat cells [25] and to the Itk-TSAd complex in murine thymocytes (Fig. 4B). In primary cells with endogenous expression of Lck, Itk and TSAd, TSAd-Itk-Lck complex formation and tyrosine phosphorylation of Itk required CXCL12 stimulation. Similarly, although Itk was phosphorylated to some extent in resting Jurkat T cells over-expressing TSAd and Itk, CXCL12 stimulation led to a further increase in Itk phosphorylation. Thus, our data indicate that CXCL12 induced phosphorylation of Itk is promoted by TSAd, at least in primary cells.

Our data from transfected 293T cells shows that Lck is required for phosphorylation of Itk Y511. Whether Lck phosphorylates Itk directly or whether Lck promotes Itk phosphorylation indirectly via activation of other kinases has not been addressed here. Heyeck and colleagues previously showed that Lck is required for phosphorylation of Itk in Sf9 insect cells [29], and here we show that TSAd strongly promotes the phosphorylation of Itk in the presence of Lck. Since both Lck and Itk may bind to TSAd, we find it likely that Lck directly phosphorylates Itk, instead of indirectly via another, as yet unidentified kinase.

The Itk Y511 phosphorylation site was essential for the increased migratory response in Jurkat T cells upon co-expression of TSAd with Itk. Whether it is phosphorylation of Itk Y511 *per se* or also the subsequent phosphorylation of Y180 in the Itk SH3 domain [42] that is important for cellular migration has not been dissected here. Phosphorylation of Y180 may also alter the ligand binding properties of Itk-SH3 as has been shown for phosphorylated versus non-phosphorylated SH3 domain of another Tec family kinase, Bruton's tyrosine kinase [43]. Thus, the outcome of the enhanced Itk tyrosine phosphorylation could be increased catalytic activity, altered ligand specificity of the Itk-SH3 domain, or both.

Mice deficient in either CXCL12 or CXCR4 show developmental defects of the vasculature, heart, brain and hematopoietic system, indicating an important role for CXCL12/CXCR4 signaling in multiple cell types [44]. One way to coordinate the effects of CXCL12 signaling in the various CXCR4-expressing cell types is to exploit tissue-specific intracellular signaling pathways. Cell specific factors, e.g. Itk, Lck, Zap-70 and SLP76, function to direct CXCR4 signaling in T cells [26], [27], [30], [45]. The observation that TSAd promotes CXCL12 signaling in T cells lends further support to this notion, since TSAd is not ubiquitously expressed in cells of hematopoietic origin (reviewed in [46]).

There appears to be extensive cross-talk between the TCR and CXCR4 pathways. However the literature is not consistent as to how molecular crosstalk between TCR and CXCR4 affects the overall cellular response when both receptors are triggered simultaneously or consecutively. While CXCR4 signaling exploits the immunoreceptor tyrosine activation motifs of the TCR [13], triggering of the TCR prior to CXCR4 stimulation, inhibits T cell migration [35], [47]. Moreover, CXCR4 and CCR5 receptors are sequestered to the immunological synapse following TCR stimulation [48] where they physically interact and co-operate to provide co-stimulatory signals after CXCL12 stimulation [49]. Obviously, crosstalk between the TCR and CXCR4 pathways may operate through the use of several of the same signaling molecules, such as Itk, Lck, Zap-70 and SLP-76. Interestingly, and in accordance with the findings of Park *et al* [25], we found that both Zap-70 and Lck were recruited to the TSAd containing Itk complex upon CXCL12 stimulation. Moreover, we have found that Zap-70 phosphorylation upon CXCL12 stimulation is affected by the presence of TSAd (Berge, unpublished data). Since TSAd is also involved in the TCR pathway [16], [18], it is possible that this molecule contributes to the crosstalk between CXCR4 and TCR, by providing spatial and temporal control of Lck and Itk. However, the net effect of TSAd on cellular function when both CXCR4 and TCR pathways are stimulated simultaneously or consecutively remains to be determined.

Most biological systems display a certain level of redundancy, in the sense that several molecules may fulfil overlapping functions. Our data clearly show that TSAd is not strictly required for CXCL12 induced actin polymerization and migration in T cells, as polymerized actin and migration can be detected also in TSAd-deficient T cells. Adapter proteins essentially function as catalysts for molecular interactions. During chemokine signalling in the absence of TSAd, other adapter proteins may take the role of TSAd. Alternatively, the interacting molecules using TSAd as an intermediary may to some extent interact also in its absence. We thus hypothesize that the physiological relevance of the TSAd-Lck-Itk complex is to enhance the signal from the ligand bound CXCR4, thus lowering the threshold for CXCL12 induced signaling. This notion is supported by our observation that at low CXCL12 doses, actin polymerization in TSAd deficient cells (lacking the TSAd-Lck-Itk complex) is attenuated, however, upon addition of higher CXCL12 doses, the requirement for TSAd and TSAd containing complexes seems to be overcome leading to F-actin levels comparable to that of TSAd containing T cells. Similarly, Zap-70 promotes CXCR4 signaling at low CXCL12 concentrations, whereas at higher doses, CXCL12 responsiveness is independent of Zap70 activity [50].

In our study, we focused on the CXCL12 chemokine since previous reports had indicated that the TSAd interaction partner Itk regulates CXCL12 signalling [26], [27]. However, as TSAd interacts with Gβ [25], which is found associated to a number of GPCRs [51], it is likely that TSAd may be a regulator of several other chemokine receptors. In support of this, our data show reduced CCL19 responsiveness in *SH2D2A* ^−/−^ thymocytes and Park *et. al.* reported that TSAd also regulates the responsiveness to RANTES [25]. We previously found that TSAd regulates VEGFR-2 induced actin polymerisation and migration in endothelial cells [24]. Moreover, very recently it was reported that TSAd may also be involved in integrin mediated migration of activated Jurkat T cells through interaction with the 67 kDa laminin binding protein (LAMR1) [52]. Thus, to what extent TSAd functions as a general adapter protein in regulating cellular migration through various signalling pathways remains to be established.

**Figure 6:**
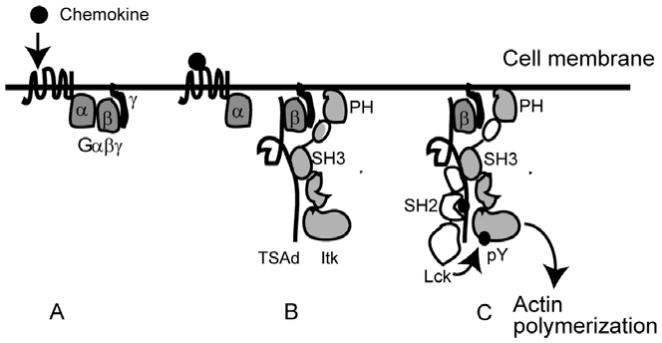
Model for TSAd regulation of CXCL12 signaling. Upon binding of CXCL12 to its G protein coupled receptor, the heterotrimeric G protein complex (A) is separated into a receptor bound Gα and a membrane bound Gβγ subunit. TSAd is recruited to Gβγ, by interacting with the Gβ subunit [25]. Similarly, Itk may also interact with the membrane via its PH domain [41] (B). By simultaneous interaction with Itk through the Itk-SH3 domain and Lck through multivalent interactions, TSAd promotes Lck mediated phosphorylation of Itk. Phosphorylated Itk becomes active, which ultimately leads to downstream actin polymerization and T cell migration (C).

In conclusion, we have shown that CXCL12 signalling is regulated by a novel molecular mechanism, which involves the interaction of TSAd with Itk. Fig. 6 depicts our current model for how TSAd contributes to CXCL12 mediated signalling. We suggest that TSAd acts as a scaffold that brings Lck and Itk in proximity to each other and to Gβγ of the activated chemokine receptor. Through its ability to interact with these kinases, TSAd primes Itk for Lck mediated phosphorylation upon CXCL12 stimulation, promoting actin polymerization and T cell migration.

## Materials and Methods

### Ethics Statement

The animals were bred under conventional conditions, regularly screened for common pathogens and housed in compliance with guidelines set by the Experimental Animal Board under the Ministry of Agriculture of Norway. The research involving breeding of transgenic animals and collection of cells from transgenic and wild type animals was approved by the The National Animal Research Authority, via their local competent person at the University of Oslo.

### Animals and murine cells

TSAd deficient (*SH2D2A* ^−/−^) mice (RIBP knockout mice) were kindly provided by Professor J. A. Bluestone, University of California [16]. *SH2D2A* ^−/−^ mice backcrossed >10 generations into C57Bl/6 were maintained on a C57Bl/6 background as described [24]. Murine thymocytes were obtained by crushing the thymus and subsequently filtering through a cell strainer (70 µm nylon, Becton Dickinson (BD) Biosciences).

### Plasmids

cDNA encoding N-terminally HA-tagged human TSAd for expression in mammalian cells has been described previously [18]. HA-tagged TSAd deletion mutants (TSAd Δex7 and TSAd Δ239–274) were generated by megaprimer PCR and subsequently cloned into the *Eco*RI site of pEF-HA. pGEX-2T-Itk-SH2, -Itk-SH3, -Itk-SH3 (W208A), pGEX-3T-Lck-SH2 and pGEX-6P-Lck-SH3 constructs were previously reported [21], [53]. For mammalian expression of C-terminally GFP tagged proteins, TSAd and TSAd Δ239–274 were subcloned into the *Hind*III/*Eco*RI and *Xho*I/*Eco*RI sites of pEGFP-N3 (Clontech), respectively. The pEF-myc-Itk and pEF-Lck constructs were kindly provided by Professor Leslie Berg and Professor Tomas Mustelin, respectively. To create Itk Y511F, pEF-myc-Itk was mutated by Quick change mutagenesis (Clontech) using custom made oligonucleotides. All PCR generated constructs were verified by sequencing.

### Antibodies and chemokines

Monoclonal antibodies (mAbs) used were anti-CD3ε (clone OKT3, American Tissue Culture Collection (ATCC)) anti-phosphotyrosine (clone 4G10), anti-Emt/Itk/Tsk (clone 2F12, Upstate Biotechnology), anti-Btk (pY551)/Itk (pY511) (BD Biosciences), anti-HA (clone HA.11, Bio Site), anti-Lck (clone IF6, a kind gift from Joseph B. Bolen), anti-GFP (clone B-2, Santa Cruz Biotechnology), anti-myc (clone 9E10, Sigma), anti-GST (clone B14, Santa Cruz Biotechnology), anti-Zap70 (clone 29, BD Biosciences), FITC-conjugated rat anti-mouse CD4 (BD Biosciences) and PE-conjugated rat anti-mouse CD8 (BD Biosciences). A polyclonal antibody against murine TSAd was generated by immunizing rabbits with a peptide representing the 20 C-terminal amino acids of murine TSAd mixed with Freund's complete adjuvans. The other polyclonal antibodies used were rabbit anti-TSAd antibodies specific for the 20 C-terminal amino acids of human TSAd [18], rabbit anti-Lck (Alexis Biochemicals), goat anti-human CXCR4 (Fusin G19, Santa Cruz Biotechnology), biotinylated goat anti-actin (I-19, Santa Cruz Biotechnology) and goat IgG (Sigma). Secondary reagents were horseradish peroxidase-conjugated goat anti-mouse IgG, goat anti-rabbit IgG or streptavidin, and FITC-conjugated donkey anti-goat IgG (Jackson ImmunoResearch Laboratories). Human and murine CXCL12 (SDF-1α) and murine CCL19 were purchased from PeproTech (Rocky Hill, NJ).

### Cell cultures and transfections

Human embryonal kidney (HEK) 293 TAg cells (here for short 293T cells) [54], Jurkat E6.1 cells (ATCC) and Jurkat TAg cells [55] were cultured in RPMI-1640 with L-glutamine supplemented with 5–10% fetal calf serum (FCS), 1 mM sodium pyruvate, 1 mM non-essential amino acids (all from GIBCOBRL®, Life Technologies™), 25 µg/ml gentamycin (Fluka, Bio Chemika) and 60 µg/ml penicillin (Alpharma). Jurkat cells were either transfected with the Amaxa nucleofector (Cell line nucleofector™ kit (#VCA-1003), program I10, 1.2×10^7^ cells and 3–9 µg DNA) or by electroporation with a BTX electroporator (Genetronix), 200 V, 70 ms, 5−10×10^6^ cells and 5–20 µg DNA in 400 µl antibiotic-free RPMI-1640 medium supplemented with 5% FCS. 293T cells (2×10^6^) were transfected with a mixture of 0,5–5 µg DNA and 25 µl Lipofectin in 5 ml Optimem 1 (GIBCOBRL®). Cells were harvested 18–24 hours after transfection.

### Cell stimulation, lysis, immunoprecipitation, GST-pull down and Western blot

Murine thymocytes or transiently transfected Jurkat T cells were resuspended in PBS at 10^8^ cells/ml, and stimulated with the indicated concentration of chemokine for the time points depicted in the figure legends. Jurkat cells (5×10^5^ cells/ml) were stimulated with 1 µg/ml anti-CD3ε in RPMI-1640 at 37°C, 5% CO_2_ for 22 hours. After stimulation, cells were lysed by addition of an equal volume of 2x lysis buffer: 0.5–2% Nonoidet P-40 (Calbiochem-Novabiochem Corporation), 0.1 M octyl-β-D-glucopyranosid, 0.1 M NaF, 20 mM Na_3_VO_4_ (all from Sigma), protease inhibitors (Protease Inhibitor Cocktail Tablets, Complete EDTA-free, Roche) in 40 mM Tris-HCl, pH 7.4, 200 mM NaCl. For immunoprecipitation, protein G Dynabeads (Dynal Biotech, Invitrogen) were precoated with relevant antibodies for 1 hr at room temperature, prior to incubation with cell lysates for 1 hr or over night at 4°C. For glutathione S-transferase (GST) pull-down analysis, GST-fusion proteins were produced in BL21 Codon plus bacteria (Stratagene) and purified on glutathione Sepharose beads (Pharmacia Biotech). Lysates from transfected 293T or Jurkat T cells were precleared three times for 1–2 hours with a 1∶1 mixture of GST-glutathion/4B Sepharose (Pharmacia Biotech). The precleared lysates were added to Itk-SH3, Itk-SH3 (W208A), Itk-SH2, Lck-SH2 or Lck-SH3-GST-gluthatione Sepharose beads and rotated for 1–2 hours at 4°C. For both immunoprecipitation and GST-pull down, the beads were washed three times with 1x lysis buffer. Proteins were eluted in reducing SDS-loading buffer, separated by SDS-PAGE and transferred to PVDF membrane (Bio Rad) using a Hoefer Semi-Phor Semi-Dry transfer unit (Amersham Biosciences).

### Chemotaxis assays

Chemotaxis of murine thymocytes was measured either using a 48-well transwell chemotaxis chamber, with 5 µm pore-sized polyvinylpyrrolidone-free filters (NeuroProbe) or using polycarbonate transwell inserts (see below). In the 48-well assay, the bottom chambers were filled with 28 µl chemotaxis medium (RPMI-1640 with 0.5% BSA and 25 mM Hepes) with or without chemokines of the indicated concentrations in triplicates. Murine cells (5×10^4^ - 1×10^5^) in 50 µl chemotaxis medium were loaded in the upper chambers. After 2 hr incubation at 37°C and 5% CO_2_, transmigrated cells in the bottom chambers were harvested and counted in a Bürker chamber. Chemotaxis was also measured using polycarbonate transwell inserts with 5 or 8 µm pores (Costar), for murine thymocytes and Jurkat T cells, respectively. Cells were washed in RPMI-1640 supplemented with 0.25% BSA and 25 mM Hepes and resuspended at 2.5×10^6^ cells/ml. 100 µl (2.5×10^5^ cells) was added to the top of the chamber (transwell) in the absence or presence of the indicated amounts of chemokine in the lower compartment. Assays were performed in duplicates or triplicates at 37°C and 5% CO_2_ for 2 hours. Cells that had passed through the filter into the lower chamber were collected, and 10 µl of an internal bead control (Bangs Laboratories, Fishers, IN) was added. The cells were counted by flow cytometry (FACS Calibur, BD Biosciences) and normalized with reference to the internal bead control.

### Actin polymerization assay

Murine thymocytes or transiently transfected Jurkat T cells (1×10^6^ cells in 100 µl PBS) were stimulated with 10–1000 ng/ml CXCL12 for the indicated time points at 37°C. After stimulation, cells were transferred to an equal volume of fixation/permeabilization/staining solution (8% formaldehyde, 0.5 mg/ml L-α-Lysophosphatidylcholin (Sigma) and 1 µg/ml FITC-Phalloidin or 1.25 U Alexa 546-phalloidin (Molecular Probes) and incubated for at least 25 minutes at room temperature. Cells were washed twice in PBS with 2% FCS and analyzed by flow cytometry (FACS Calibur, BD Biosciences).

### Statistics

Experiments were performed at least three times. Results were compared using two tailed paired Student's t test. P values <0,05 were considered significant.
